# Small Universal Bacteria and Plasmid Computing Systems

**DOI:** 10.3390/molecules23061307

**Published:** 2018-05-29

**Authors:** Xun Wang, Pan Zheng, Tongmao Ma, Tao Song

**Affiliations:** 1 College of Computer and Communication Engineering, China University of Petroleum, Qingdao 266580, China; wangsyun@upc.edu.cn (X.W.); matongmao@163.com (T.M.); 2 Department of Accounting and Information Systems, University of Canterbury, Christchurch 8041, New Zealand; panzheng@ieee.org; 3 Departamento de Inteligencia Artificial, Universidad Politcnica de Madrid (UPM), Campus de Montegancedo, 28660 Boadilla del Monte, Spain

**Keywords:** bacterial computing, bacteria and plasmid system, Turing universality, recursively enumerable function

## Abstract

Bacterial computing is a known candidate in natural computing, the aim being to construct “bacterial computers” for solving complex problems. In this paper, a new kind of bacterial computing system, named the bacteria and plasmid computing system (BP system), is proposed. We investigate the computational power of BP systems with finite numbers of bacteria and plasmids. Specifically, it is obtained in a constructive way that a BP system with 2 bacteria and 34 plasmids is Turing universal. The results provide a theoretical cornerstone to construct powerful bacterial computers and demonstrate a concept of paradigms using a “reasonable” number of bacteria and plasmids for such devices.

## 1. Introduction

In cell biology, bacteria, despite their simplicity, contain a well-developed cell structure that is responsible for some of their unique biological structures and pathogenicity. The bacterial DNA resides inside the bacterial cytoplasm, for which transfer of cellular information, transcription, and DNA replication occurs within the same compartment [1,2]. Along with chromosomal DNA, most bacteria also contain small independent pieces of DNA called plasmids, which can be conveniently obtained and released by a bacterium to act as a gene delivery vehicle between bacteria in the form of horizontal gene transfer [3].

Bacterial computing was coined with the purpose of building biological machines, which are developed to solve real-life engineering and science problems [4]. Practically, bacterial computing proves mechanisms and the possibility of using bacteria for solving problems in vivo. If an individual bacterium can perform computation work as a computer, this envisions a way to build millions of computers in vivo. These “computers”, combined together, can perform complicated computing tasks with efficient communication via plasmids. Using such conjugation, DNA molecules, acting as information carriers, can be transmitted from one cell to another. On the basis of the communication, information in one bacteria can be moved to another and can be used for further information processing [5,6].

Bacterial computing models belong to the field of bio-computing models, such as DNA computing models [7,8,9] and membrane computing models [10,11,12]. Because of the computational intelligence and parallel information processing strategy in biological systems, most of the bio-computing models have been proven to have the desired computational power. Most of these can do what a Turing machine can do (see, e.g., [13,14,15,16,17,18,19]). The proposed bacterial computing models can provide powerful computing models at the theoretical level but a lack of practical results. Current bacterial computing models are designed for solving certain specific biological applications, such as bacteria signal pathway detecting, but give no result for computing power analysis.

**Figure 1:**
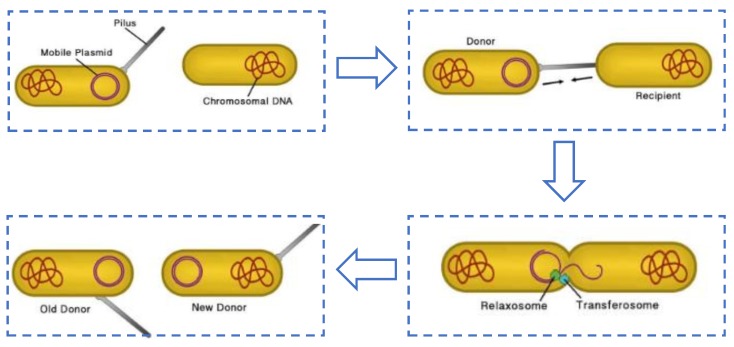
Bacteria conjugation from biological point of view.

In general bacterial computing models, information to be processed is encoded by DNA sequences, and conjugation is the tool for communicating among bacteria. The biological process is shown in Figure 1.

Looking for small universal computing devices, such as small universal Turing machines [20,21], small universal register machines [22], small universal cellular automata [23], small universal circular Post machines [24], and so on, is a natural and well-investigated topic in computer science. Recently, this topic started to be considered also in the framework of bio-computing models [25,26,27,28,29,30,31].

In this work, we focus on designing small universal bacteria and plasmid computing systems (BP systems); that is, we construct Turing universal BP systems with finite numbers of bacteria and plasmids. Specifically, we demonstrate that a BP system with 2 bacteria and 34 plasmids is universal for computing recursively enumerable functions and families of sets of natural numbers. In the universality proofs, 2 bacteria are sufficient, as in [32], but the numbers of plasmids needed are reduced to about 10 from a possible infinite number. The results provide a theoretical cornerstone to construct powerful “bacterial computers” and demonstrate a concept of paradigms using a “reasonable” number of bacteria and plasmids for these devices.

## 2. The Bacteria and Plasmid System

In this work, as for automata in automata theory, the BP system is formally designed and defined. In general, the system is composed of three main components:

– a set of bacteria;
– a set of plasmids;
– a set of evolution rules in each bacterium, including conjugation rules and gene-editing (inserting/deleting) rules.

The evolution rules are in the form of productions in formal language theory, which are used to process and communicate information among bacteria. Such a system is proven to be powerful for a number of computing devices; that is, they can compute the sets of natural numbers that are Turing computable. However, in the universality proof, the number of plasmids involved is not limited. It is possible to use an infinite number of plasmids for information processing and exchanging. Such a feature is acceptable (as for the infinite tape in Turing machines) in mathematic theory but is not feasible with the biological facts.

A BP system of degree *m* is a construct of the following form:

$$\Pi =(O,b_{1},b_{2},\ldots ,b_{m},P,b_{out}),\mathrm{where}\mathrm{the}\mathrm{following}\mathrm{are}\mathrm{true}.$$

- $O=\{g_{1},g_{2},\ldots ,g_{n}\}$ is a set of genes in the chromosomal DNA of bacteria.
- $P=P_{crispr}\cup P_{temp}\cup \left\{p_{null}\right\}$ is a set of plasmids.
  – Plasmids in $P_{crispr}$ are of the form $\left(cas9,gRNA_{g_{i}}^{\alpha }\right)$ with α∈{insert,delete}, which is used for cutting specific genes.
  – Plasmids in $P_{temp}$ are of the form $\left(gRNA_{g_{i}}^{template}\right)$, which takes templates of genes to be inserted.
  – Plasmid $p_{null}$ is of the form $\left(Pro_{Rap}^{Rel}\right)$ for bacteria conjugation.
- Variables $b_{1},b_{2},\ldots ,b_{m}$ are *m* bacteria of the form $b_{i}=\left(w_{i},R_{i}\right)$, where
  – $w_{i}$ is a set of genes over *O* initially placed in bacterium $b_{i}$;
  – $R_{i}$ is a set of rules in bacterium $b_{i}$ of the following forms:
    (1) **Conjugation rule** is of the form $\left(\mathrm{ATP}-P_{c},b_{i}/b_{j},\mathrm{ATP}-P_{c}^{\prime }\right)$, by which ATP in bacterium $b_{i}$ is consumed and a set of plasmids $P_{c}^{\prime }\subseteq P$ associated with ATP is transmitted into bacterium $b_{j}$.
    (2) **CRISPR/Cas9 gene inserting rule** is of the form $p_{i}p_{s_{i}}\times \left(g_{j},g_{k}\right)$, where $p_{i}\in P_{crispr}$, α=insert, $p_{s_{i}}\in P_{temp}$, and $g_{j}$ and $g_{k}$ are two neighboring genes. The insertion is operated if and only if $g_{j}$ and $g_{k}$ are neighboring genes and plasmids $p_{i}p_{s_{i}}$ are present in the bacterium.
    (3) **CRISPR/Cas9 gene deleting rule** is of the form $p_{i}\times \left(g_{j},g_{k}\right)$ with $p_{i}p_{null}\in P_{crispr}$, α=detele, and $g_{j}$ and $g_{k}$ being two neighboring genes. The rule can be used if and only there exists gene $g_{i}$ placed between the two neighboring genes.
- Variable $b_{out}$ is the output bacterium.

It is possible to have more than one enabled conjugation rule at a certain moment in a bacterium, but only one is non-deterministically chosen for use. This is due to the biological fact that ATP can support the transmission of one plasmid but not all of the plasmids. If a bacterium has more than one CRISPR/Cas9 operating rule associated with a certain common plasmid, only one of the rules is non-deterministically chosen for use; if the enabled CRISPR/Cas9 operating rules are associated with different plasmids, all of them will be used to edit the related genes.

The configuration of the system is described by chromosomal DNA encoding the information in each bacterium. Thus, the initial configuration is $\langle (w_{1},w_{2},\ldots ,w_{m}\rangle$. Using the conjugation and CRISPR/Cas9 rules defined above, we can define the transitions among configurations. Any sequence of transitions starting from the initial configuration is called a computation. A computation is called successful if it reaches a halting configuration, that is, no rule can be used in any bacterium. The computational result is encoded by the chromosomal DNA in bacterium $b_{out}$ when the system halts, where $b_{out}\in \left\{b_{1},b_{2},\ldots ,b_{m}\right\}$ denotes the output bacterium. There are several ways to encode numbers by the chromosomal DNA. We use the number of genes in the chromosomal DNA to encode different numbers computed by the system.

The set of numbers computed by system Π is denoted by N(Π). We denote by $NBP(bact_{j},plas_{k})$ the family of sets of numbers computed/generated by BP systems with *m* bacteria and *k* plasmids (if no limit is imposed on the values of parameters *m* and *k*, then the notation is replaced by *).

We need an input bacterium to receive genetic signals in the form of short DNA segments from the environment or certain bacteria, as well as an output bacteria, with which the system can compute functions. The input bacterium is denoted by $b_{in}$ with $b_{in}\in \left\{b_{1},b_{2},\ldots ,b_{m}\right\}$. Input bacterium $b_{in}$ can read/receive information from the environment, where information is encoded by DNA segments or a string of genes. When a BP system has both input and output bacteria, it starts by reading/receiving information from the environment through input bacterium $b_{in}$. After reading the input information, the system starts its computation by using the conjugation and CRISPR/Cas9 gene inserting/deleting rules; it then finally halts. The computational result is stored in the output bacterium $b_{out}$ encoded by a number of certain genes.

Mathematically, if the input information is *x*, which is encoded by DNA segments composed of *x* genes, when the system halts, bacterium $b_{out}$ holds *y* genes. It is said that the BP system can compute the function f(x)=y. In general, if the inputs are $x_{1},x_{2},\ldots ,x_{n}$ in the form of DNA strands containing $x_{i}$ copies of gene $g_{i}$ with i=1,2,…,n, when the system halts, we obtain the computational result *y* in the output bacterium in the form of *y* copies of genes. The system is said to compute the function $f(x_{1},x_{2},\ldots ,x_{n})=y$.

## 3. Universality Results

In this section, we construct two small universal BP systems. Specifically, we construct a Turing universal BP system with 2 bacteria and 34 plasmids to compute recursively enumerable functions. As a natural-number computing device, a universal BP system with 2 bacteria and 34 plasmids is achieved.

In the following universality proofs, the notion of a register machine is used. A register machine is a construct of the form $M=(m,H,l_{0},l_{h},R)$, where *m* is the number of registers, *H* is the set of instruction labels, $l_{0}$ is the start label, $l_{h}$ is the halt label (assigned to instruction HALT), and *R* is the set of instructions; each label from *H* labels only one instruction from *R*, thus precisely identifying it. The instructions are of the following forms:

- $l_{i}:($ADD$\left(r\right),l_{j},l_{k})$ (add 1 to register *r* and then go to one of the instructions with labels $l_{j}$ and $l_{k}$);
- $l_{i}:($SUB$\left(r\right),l_{j},l_{k})$ (if register *r* is non-zero, then subtract 1 from it, and go to the instruction with label $l_{j}$; otherwise, go to the instruction with label $l_{k}$);
- $l_{h}:$ HALT (the halt instruction).

A register machine *M* generates a set N(M) of numbers in the following way: it starts with all registers being empty (i.e., storing the number zero) and then applies the instruction with label $l_{0}$; it continues to apply instructions as indicated by the labels (and made possible by the contents of registers). If the register machine finally reaches the halt instruction, then the number *n* present in specified register 0 at that time is said to be generated by *M*. If the computation does not halt, then no number is generated. It is known (e.g., see [33]) that register machines generate all sets of numbers that are Turing computable.

A register machine can also compute functions. In [22], register machines are proposed for computing functions, with the universality defined as follows: Let $\varphi_{x}\left(y\right)$ be a fixed admissible enumeration of the unary partial recursive functions. A register machine *M* is said to be universal if there is a recursive function *g* such that for all natural numbers *x* and *y*, it holds $\varphi_{x}\left(y\right)=M\left(g\left(x\right),y\right)$; that is, with input g(x) and *y* introduced in registers 1 and 2, the result $\varphi_{x}\left(y\right)$ is obtained in register 0 when *M* halts.

**Figure 2:**
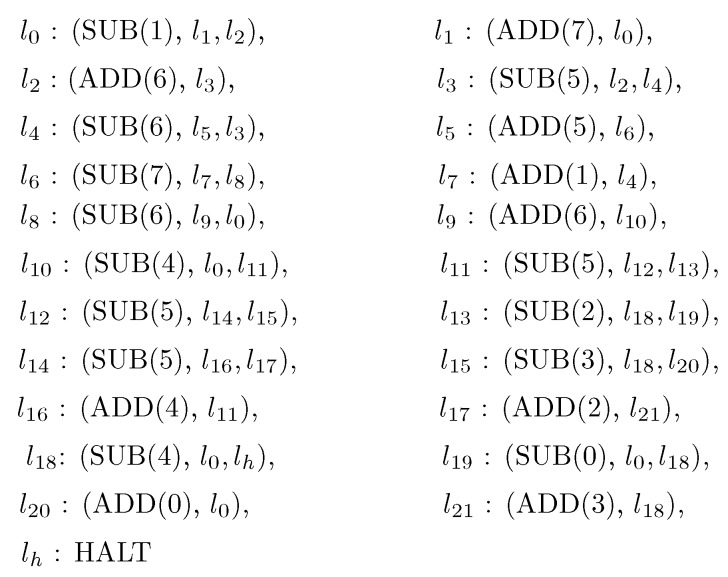
The universal register machine for computing Turing-computable functions [22].

A specific universal register machine $M_{u}$ shown in Figure 2 is used here, which was modified by a universal register machine from [22]. Specifically, the universal register machine from [22] contains a separate check for zero of register 6 of the form $l_{8}:$ (SUB(6), $l_{0},l_{10}$); this instruction was replaced in $M_{u}$ by $l_{8}:$ (SUB(6), $l_{9},l_{0})$, $l_{9}:$(ADD(6), $l_{10})$ (see Figure 2). Therefore, in the modified universal register machine, there are 8 registers (numbered from 0 to 7) and 23 instructions (hence 23 labels), the last instruction being the halting instruction. The input numbers are introduced in registers 1 and 2, and the result is obtained in register 0.

### 3.1. A Small Universal BP System as Function Computing Device

**Theorem** **1.** *There exists a Turing universal BP system with 2 bacteria and 34 plasmids that can compute Turing-computable recursively enumerable functions.*

**Proof.**  To this aim, we construct a BP system Π with 2 bacteria and 34 plasmids to simulate the register machine $M_{u}$ shown in Figure 2. The system Π is of the following form:

$$\Pi =(O,b_{1},b_{2},P,b_{in},b_{out}),\mathrm{where}\;\mathrm{the}\;\mathrm{following}\;\mathrm{are}\;\mathrm{true}.$$

- $O=\{g_{0},g_{1},\ldots ,g_{7},g_{m}\}$ is set of genes in chromosomal DNA of bacteria.
- $P=P_{crispr}\cup P_{temp}\cup \left\{p_{null}\right\}$ is a set of plasmids shown in Table 1, where
  – $P_{crispr}=\left\{p_{1},p_{2},\ldots ,p_{22},p_{h}\right\}$, whose elements associated with the labels of instructions are used for gene cutting;
  – $P_{temp}=\left\{p_{s_{i}}|i=1,2,5,7,9,16,17,20,21\right\}$ are plasmids taking templates of genes to be inserted, which are used for simulating ADD instructions;
  – plasmid $p_{null}$ for bacteria conjugation is used for simulating SUB instructions.
- $b_{1}=\left(w_{1},R_{1}\right)$, where $w_{1}=\lambda$, meaning no initial chromosomal DNA is placed in bacteria $b_{1}$; the set of rules $R_{1}$ is shown in Table 2.
- $b_{2}=\left(w_{2},R_{2}\right)$, where $w_{2}=g_{0}g_{m}g_{1}g_{m}g_{2}g_{m}g_{3}g_{m}g_{4}g_{m}g_{5}g_{m}g_{6}g_{m}g_{7}g_{m}$, indicating the initially placed chromosomal DNA in bacterium $b_{2}$; the set of rules $R_{2}$ is shown in Table 2.
- $b_{in}=b_{out}=b_{2}$, which means bacterium $b_{2}$ can read signals from the environment, and when the system halts, the computational result is stored in bacterium $b_{2}$.

In general, for each add instruction $l_{i}$ acting on register r∈{0,1,2,3,4,5,6,7}, plasmids $p_{i}=\left(cas9,gRNA_{g_{r}}^{insert}\right)$ and $p_{s_{i}}=\left(gRNA_{g_{r}}^{template}\right)$ are associated; for any SUB instruction $l_{i}$ acting on register r∈{0,1,2,3,4,5,6,7}, a plasmid $p_{i}=\left(cas9,gRNA_{g_{r}}^{delete}\right)$ is associated in system Π. The numbers stored in register *r* are encoded by the number of copies of gene $g_{r}$ with r∈{0,1,2,3,4,5,6,7} in chromosomal DNA of bacterium $b_{2}$. Specifically, if the number stored in register *r* is n≥0, then bacterium $b_{2}$ contains n+1 copies of gene $g_{r}$.

During the simulation of register machine $M_{u}$ by system Π, when bacterium $b_{1}$ holds a pair of plasmids $p_{i}p_{s_{i}}$ (respectively $p_{i}p_{null}$) and ATP, the system starts to simulate an ADD instruction (respectively a SUB instruction) $l_{i}$ of $M_{u}$: plasmids $p_{i}p_{s_{i}}$ (respectively $p_{i}p_{null}$) are transmitted to bacterium $b_{2}$ by the conjugation rule; then one copy of gene $g_{r}$ between neighboring genes $g_{r}$ and $g_{m}$ is inserted (respectively deleted) to simulate increasing (respectively decreasing) the number in register *r* by 1; after this, bacterium $b_{2}$ sends ATP and plasmids $p_{j}p_{null}$ to bacterium $b_{1}$ if the proceeding instruction $l_{j}$ is a SUB instruction or plasmids $p_{j}p_{s_{j}}$ if the proceeding $l_{j}$ is an ADD instruction.

Initially, there is no chromosomal DNA initially placed in bacterium $b_{1}$, but bacterium $b_{2}$ has genes $w_{2}=g_{0}g_{m}g_{1}g_{m}g_{2}g_{m}g_{3}g_{m}g_{4}g_{m}g_{5}g_{m}g_{6}g_{m}g_{7}g_{m}$. At the beginning, the system receives g(x) copies of gene $g_{1}$ and *y* copies of gene $g_{2}$ from the environment through input bacterium $b_{2}$, which simulates the numbers g(x) and *y* being introduced in registers 1 and 2 for register machine $M_{u}$. In this way, the chromosomal DNA of bacterium $b_{2}$ becomes

$$g_{0}g_{m}g_{1}^{g(x)+1}g_{m}g_{2}^{y+1}g_{m}g_{3}g_{m}g_{4}g_{m}g_{5}g_{m}g_{6}g_{m}g_{7}g_{m}.$$

**Figure 3:**
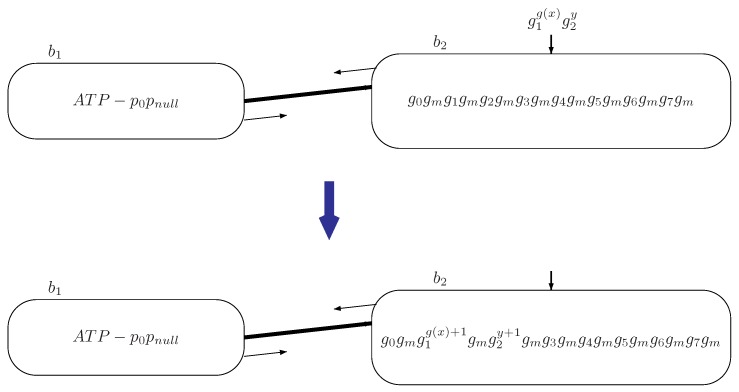
The transition of system Π by reading input information encoded by g(x) copies of genes $g_{1}$ and *y* copies of gene $g_{2}$ through input bacterium $b_{2}$.

Once completing the reading of information from the environment, a pair of plasmids $p_{0}p_{s_{0}}$ and one unit of ATP is placed in bacterium $b_{1}$ to trigger the computation; meanwhile no plasmid or ATP is initially contained in bacterium $b_{2}$. The transition of system Π by reading input signals encoded by g(x) copies of genes $g_{1}$ and *y* copies of gene $g_{2}$ through input bacterium $b_{2}$ is shown in Figure 3.

In what follows, we explain how system Π simulates ADD instructions and SUB instructions and outputs the computational result.

**Simulating the ADD instruction:**$l_{i}:$ (DD(r), $l_{j})$.

We assume at a certain moment that system Π starts to simulate an ADD instruction $l_{i}$ of $M_{u}$, acting on register r∈{0,1,2,…,7}. At that moment, bacterium $b_{1}$ holds two plasmids $p_{i}p_{s_{i}}$ and ATP, such that the conjugation rule $\left(\mathrm{ATP}-p_{i}p_{s_{i}},b_{1}/b_{2},\mathrm{ATP}-p_{i}p_{s_{i}}\right)$ is used. By using the conjugation rule, plasmids $p_{i}p_{s_{i}}$ and ATP are transmitted to bacterium $b_{2}$. In system Π, plasmids $p_{i}$ and $p_{s_{i}}$ are associated with the ADD instruction $l_{i}$, where plasmid $p_{i}$ is of the form $p_{i}=\left(cas9,gRNA_{g_{r}}^{insert}\right)$ for cutting a certain site of chromosomal DNA, and $p_{s_{i}}$ is of the form $p_{i}=\left(gRNA_{g_{r}}^{template}\right)$ carrying the gene to be inserted.

In bacterium $b_{2}$, the CRISPR/Cas9 inserting rule $p_{i}\times \left(g_{r},g_{m}\right)$ is used to insert gene $g_{r}$ between neighboring genes $g_{r}$ and $g_{m}$. In this way, the number of gene $g_{r}$ of bacterium $b_{2}$ is increased by 1, which simulates the number in register *r* being increased by 1. We note that there is a unique position at which gene $g_{r}$ can be inserted with the context of neighboring $g_{r}$ and $g_{m}$.

By using the CRISPR/Cas9 inserting rule, plasmid $p_{i}$ is consumed, and plasmid $p_{s_{i}}$ and ATP remain in bacterium $b_{2}$. The conjugation rule in bacterium $b_{2}$ is designed by the operation of the proceeding instruction $l_{j}$. One of the following two cases occurs in bacterium $b_{2}$.

- If instruction $l_{j}$ is an ADD instruction, then bacterium $b_{2}$ has the conjugation rule $\left(\mathrm{ATP}-p_{si},b_{2}/b_{1},\mathrm{ATP}-p_{j}p_{s_{j}}\right)$. By using the rule, plasmids $p_{j}p_{s_{j}}$ and ATP are conjugated to bacterium $b_{1}$. In this case, system Π starts to simulate the proceeding ADD instruction $l_{j}$.
- If instruction $l_{j}$ is a SUB instruction, then bacterium $b_{2}$ has the conjugation rule $\left(\mathrm{ATP}-p_{si},b_{2}/b_{1},\mathrm{ATP}-p_{j}p_{null}\right)$, by which plasmids $p_{j}p_{null}$ and ATP are transmitted to bacterium $b_{1}$. In this case, system Π starts to simulate the proceeding SUB instruction $l_{j}$.

Therefore, system Π can correctly simulate the ADD instruction of $M_{u}$. The system starts from bacterium $b_{1}$ having plasmid $p_{i}p_{s_{i}}$ and ATP, which are transmitted to bacterium $b_{2}$ by the conjugation rule. In bacterium $b_{2}$, the number of gene $g_{r}$ in chromosomal DNA is increased by 1 using the CRISPR/Cas9 gene inserting rule, and plasmids $p_{j}p_{s_{j}}$ (if the proceeding instruction $l_{j}$ is an ADD instruction) or $p_{j}p_{null}$ (if the proceeding instruction $l_{j}$ is a SUB instruction) are transmitted to bacterium $b_{1}$, which means that system Π starts to simulate instruction $l_{j}$.

**Simulating the SUB instruction:**$l_{i}:$ (SUB(r), $l_{j},l_{k})$.

We suppose at a certain computation step that system Π has to simulate a SUB instruction $l_{i}:$ (SUB(r), $l_{j},l_{k})$. For any SUB instruction $l_{i}$, plasmid $p_{i}$ of the form $p_{i}=\left(cas9,gRNA_{g_{r}}^{delete}\right)$ is associated in system Π. In bacterium $b_{1}$, there are plasmids $p_{i}p_{null}$ and ATP such that the conjugation rule $\left(\mathrm{ATP}-p_{i}p_{null},b_{1}/b_{2},\mathrm{ATP}-p_{i}p_{null}\right)$ can be used. In bacterium $b_{2}$, it has the following two cases.

– If there is at least one gene $g_{r}$ existing between neighboring genes $g_{r}$ and $g_{m}$ in chromosomal DNA of bacterium $b_{2}$ (corresponding to the case that the number stored in register *r* is n>0), then the CRISPR/Cas9 deleting rule $p_{i}\times \left(g_{1},g_{m}\right)$ is used to delete one copy of gene $g_{r}$ from chromosomal DNA. This simulates the number stored in register *r* being decreased by 1. By consuming plasmid $p_{i}$, bacterium $b_{2}$ retains plasmid $p_{null}$ and ATP such that a conjugation rule $\left(\mathrm{ATP}-p_{null},b_{2}/b_{1},\mathrm{ATP}-p_{j}p_{s_{j}}\right)$ or $\left(\mathrm{ATP}-p_{null},b_{2}/b_{1},\mathrm{ATP}-p_{j}p_{null}\right)$ is used, which depends on whether the proceeding instruction would be an ADD or a SUB instruction. In this way, plasmids $p_{j}p_{s_{j}}$ or $p_{j}p_{null})$ and ATP are transmitted to bacterium $b_{1}$. The system starts to simulate instruction $l_{j}$.
– If there is no gene $g_{r}$ existing between neighboring genes $g_{r}$ and $g_{m}$ in chromosomal DNA of bacterium $b_{2}$ (corresponding to the case that the number stored in register *r* is 0), then the CRISPR/Cas9 deleting rule $p_{i}\times \left(g_{1},g_{m}\right)$ cannot be used, but a conjugation rule $\left(\mathrm{ATP}-p_{i}p_{null},b_{2}/b_{1},\mathrm{ATP}-p_{k}p_{s_{k}}\right)$ or $\left(\mathrm{ATP}-p_{i}p_{null},b_{2}/b_{1},\mathrm{ATP}-p_{k}p_{null}\right)$ is able to be used. Plasmids ($p_{k}p_{s_{k}}$ or $p_{k}p_{null}$) and ATP are conjugated to bacterium $b_{1}$, which means the system starts to simulate instruction $l_{k}$.

We note that when plasmids $p_{i}p_{null}$ are conjugated to bacterium $b_{2}$ from bacterium $b_{1}$, it may happen that both the CRISPR/Cas9 deleting rule $p_{i}\times \left(g_{1},g_{m}\right)$ and $\left(\mathrm{ATP}-p_{i}p_{null},b_{2}/b_{1},\mathrm{ATP}-p_{k}p_{s_{k}}\right)$ (or $\left(\mathrm{ATP}-p_{i}p_{null},b_{2}/b_{1},\mathrm{ATP}-p_{k}p_{null}\right)$) can be used. In this case, the CRISPR/Cas9 deleting rule $p_{i}\times \left(g_{1},g_{m}\right)$ will be applied because of the fact that it has priority over the plasmid transferring rule.

The simulation of a SUB instruction is correct: System Π starts from bacterium $b_{1}$ having plasmid $p_{i}p_{null}$ and ATP and ends with plasmid $p_{j}p_{s_{j}}$ or $p_{j}p_{null}$ and ATP (if the number stored in register *r* is n>0) to start the simulation of instruction $l_{j}$; otherwise it ends with plasmid $p_{k}p_{s_{k}}$ or $p_{k}p_{null}$ and ATP (if the number stored in register *r* is 0) to start the simulation of instruction $l_{k}$.

**Simulating the halt instruction:**$l_{h}:$ HALT.

When register machine $M_{u}$ reaches the halt instruction $l_{h}:$ HALT, the computation of register machine $M_{u}$ halts. At that moment, bacterium $b_{1}$ in system Π holds plasmids $p_{h}p_{s_{h}}$ and ATP, and the conjugation rule $\left(\mathrm{ATP}-p_{h}p_{sh},b_{1}/b_{2},\mathrm{ATP}-p_{h}p_{s_{h}}\right)$ can be used. By using the rule, plasmids $p_{h}p_{s_{h}}$ and ATP are transmitted to bacterium $b_{2}$; no gene can be edited by plasmid $p_{h}$, and no rule can be used. Hence, the computation of system Π finally halts.

The number of gene $g_{0}$ in chromosomal DNA of bacterium $b_{2}$ encodes the number stored in register 0 of $M_{u}$. If the number stored in register 0 is n>0, then there are n+1 copies of gene $g_{0}$ in chromosomal DNA of bacterium $b_{2}$. The computational result can be obtained by counting the number of gene $g_{0}$ in chromosomal DNA of bacterium $b_{2}$.

From the above description of system Π and its work, it is clear that system Π can simulate each computation of $M_{u}$. We can check that the constructed system Π has

- 2 bacterium for conjugation with each other;
- 22 plasmids $p_{i}$ for the 22 ADD and SUB instructions with i=0,1,2,…21;
- 9 plasmids $p_{s_{i}}$ for 9 ADD instructions with i=1,2,5,7,9,16,17,20,21;
- 1 plasmid $p_{null}$ for the 13 SUB instructions;
- 2 plasmids $p_{h}$ and $p_{s_{h}}$ for the HALT instruction;
- 8 genes $g_{i}$ for encoding numbers in registers *i* with i=0,1,2…7;
- 1 gene $g_{m}$ for separating gene $g_{i}$ in chromosomal DNA.

This gives, in total, 2 bacteria, 34 plasmids, and 9 genes.

This concludes the proof. ☐

### 3.2. A Small Universal BP System as a Number Generator

In this section, we construct a small universal BP system as a number generator. A BP system $\Pi_{u}$ is universal if, given a fixed admissible enumeration of the unary partial recursive functions $\left(\varphi_{0},\varphi_{1},\ldots \right)$, there is a recursive function *g* such that for each natural number *x*, whenever we input the number g(x) in $\Pi_{u}$, the set of numbers generated by the system is equal to {$n\in N|\varphi_{x}\left(n\right)$ is defined}. In other words, after introducing the “code” g(x) of the partial recursive function $\varphi_{x}$ in the form of g(x) copies of certain genes in chromosomal DNA of the input bacterium, the BP system generates all numbers *n* for which $\varphi_{x}\left(n\right)$ is defined.

System $\Pi_{u}$ has the same topological structure, plasmids, and evolution rules as system Π constructed in Section 3.1, but the input bacterium is $b_{2}$ and the output bacterium is $b_{1}$. Differently from the universal computing devices considered in Section 3.1, the strategy to simulate a universal register machine as a number generator is as follows.

**Step 1.** The output bacterium $b_{1}$ initially has *n* copies of gene $g_{m}$.

**Step 2.** System $\Pi_{u}$ starts by loading g(x) copies of gene $g_{1}$ and *n* copies of gene $g_{2}$ in the input bacterium $b_{2}$.

**Step 3.** The computation of $\Pi_{u}$ is activated by using plasmid $p_{0}p_{null}$ to simulate the register machine $M_{u}$ from Figure 2, with g(x) stored in register 1, and number *n* stored in register 2.

If the computation in register machine $M_{u}$ halts, instruction $\sigma_{l_{h}}$ can finally be activated. To simulate register machine $M_{u}$ reaching the HALT instruction, system $\Pi_{u}$ holds plasmids $p_{h}p_{s_{h}}$ and transmits them to bacterium $b_{2}$. After this, system $\Pi_{u}$ halts, as no rule can be used in bacterium $b_{2}$. When the system halts, the number of gene $g_{m}$ in the output bacterium $b_{1}$ is the computational result, which is exactly the number *n*. Hence, the number *n* can be computed/generated by system $\Pi_{u}$.

**Figure 4:**
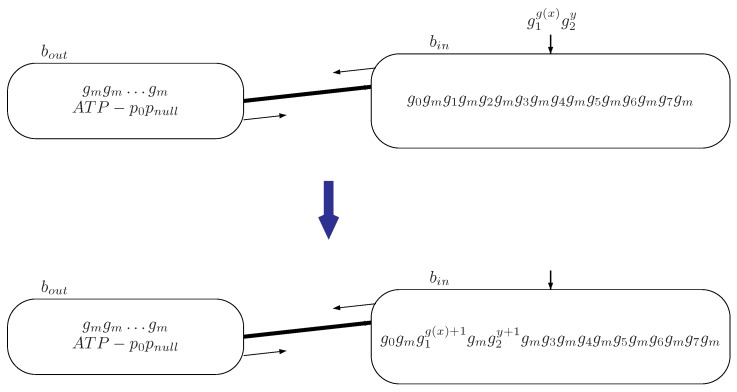
The initial configuration and transition of system $\Pi_{u}$ by reading input information encoded by g(x) copies of genes $g_{1}$ and *n* copies of gene $g_{2}$ through input bacterium $b_{2}$.

The difference between systems Π and $\Pi_{u}$ is the loading input information process. The initial configuration and transition of system $\Pi_{u}$ by reading input signals encoded by g(x) copies of genes $g_{1}$ and *n* copies of gene $g_{2}$ through input bacterium $b_{2}$ are shown in Figure 4.

We can check that the constructed system $\Pi_{u}$ has

- 2 bacterium for the conjugation with each other;
- 22 plasmids $p_{i}$ for the 22 ADD and SUB instructions with i=0,1,2,…21;
- 9 plasmids $p_{s_{i}}$ for 9 ADD instructions with i=1,2,5,7,9,16,17,20,21;
- 1 plasmid $p_{null}$ for the 13 SUB instructions;
- 2 plasmids $p_{h}$ and $p_{s_{h}}$ for the HALT instruction;
- 8 genes $g_{i}$ for encoding numbers in registers *i* with i=0,1,2…7;
- 1 gene $g_{m}$ for separating gene $g_{i}$ in chromosomal DNA.

This gives, in total, 2 bacteria, 34 plasmids, and 9 genes.

Therefore, we have the following theorem.

**Theorem** **2.** *There is a Turing universal BP system with 2 bacteria and 34 plasmids that can compute a Turing-computable set of natural numbers.*

## 4. Conclusions

In this work, we construct two small universal BP systems. Specifically, it is obtained that a BP system with 2 bacteria, 34 plasmids, and 9 genes is universal for both computing recursively enumerable functions and computing/generating a family of sets of natural numbers. It is obtained that 34 plasmids are sufficient for constructing Turing universal BP systems. This provides theoretical support as well as paradigms using a reasonable number of bacteria and plasmids to construct powerful bacterial computers.

Following the research line, finding smaller universal BP systems deserves further research. A possible way to slightly decrease the number of plasmids used in small universal BP systems is using code optimization, exploiting some particularities of the register machine $M_{u}$. For example, as considered in [25], for the sequence of two consecutive ADD instructions $l_{17}$: (ADD$\left(2\right),l_{21})$ and $l_{21}$: (ADD$\left(3\right),l_{18})$, without any other instruction addressing the label $l_{21}$, the two ADD modules can be combined. However, a challenging problem regards what the minimum size of a universal BP system is—in other words, what the borderline between universality and non-universality is. Characterization of universality by BP systems is expected. A balance between the number of bacteria and plasmids in universal BP systems can be considered, that is, using more bacteria to reduce the number of plasmids.

It is worth developing the applications of BP systems. Bio-inspiring computing models perform well in computations, particularly in solving computational complex problems in feasible time [34,35,36]. It is of interest to use BP systems to solve computationally hard problems. Some specific applications using BP systems would be of interest to researchers from biological fields.

In artificial intelligence, there are many bio-inspired algorithms (see, e.g., [37,38]). It is worth designing bacteria-computing-inspired algorithms or introducing bacteria computing operators in classical algorithms. Additionally, it would be meaningful to construct powerful bacterial computers or computing devices in biological labs.

## Figures and Tables

**Table 1:** Plasmids in system Π.

Plasmid	Forms of Plasmids	Plasmid	Forms of Plasmids
$p_{0}$	$p_{0}=\left(cas9,gRNA_{g_{1}}^{delete}\right)$	$p_{16}$	$p_{16}=\left(cas9,gRNA_{g_{4}}^{insert}\right)$
$p_{1}$	$p_{1}=\left(cas9,gRNA_{g_{7}}^{insert}\right)$	$p_{17}$	$p_{17}=\left(cas9,gRNA_{g_{2}}^{insert}\right)$
$p_{2}$	$p_{2}=\left(cas9,gRNA_{g_{6}}^{insert}\right)$	$p_{18}$	$p_{18}=\left(cas9,gRNA_{g_{4}}^{delete}\right)$
$p_{3}$	$p_{3}=\left(cas9,gRNA_{g_{5}}^{delete}\right)$	$p_{19}$	$p_{19}=\left(cas9,gRNA_{g_{3}}^{delete}\right)$
$p_{4}$	$p_{4}=\left(cas9,gRNA_{g_{6}}^{delete}\right)$	$p_{20}$	$p_{20}=\left(cas9,gRNA_{g_{0}}^{insert}\right)$
$p_{5}$	$p_{5}=\left(cas9,gRNA_{g_{5}}^{insert}\right)$	$p_{21}$	$p_{21}=\left(cas9,gRNA_{g_{3}}^{insert}\right)$
$p_{6}$	$p_{6}=\left(cas9,gRNA_{g_{7}}^{delete}\right)$	$p_{h}$	$p_{h}=\left(cas9,gRNA_{g_{h}}^{delete}\right)$
$p_{7}$	$p_{7}=\left(cas9,gRNA_{g_{1}}^{insert}\right)$	$p_{s_{1}}$	$p_{s_{1}}=\left(gRNA_{g_{7}}^{template}\right)$
$p_{8}$	$p_{8}=\left(cas9,gRNA_{g_{6}}^{delete}\right)$	$p_{s_{2}}$	$p_{s_{2}}=\left(gRNA_{g_{6}}^{template}\right)$
$p_{9}$	$p_{9}=\left(cas9,gRNA_{g_{6}}^{insert}\right)$	$p_{s_{5}}$	$p_{s_{5}}=\left(gRNA_{g_{5}}^{template}\right)$
$p_{10}$	$p_{10}=\left(cas9,gRNA_{g_{4}}^{delete}\right)$	$p_{s_{7}}$	$p_{s_{7}}=\left(gRNA_{g_{1}}^{template}\right)$
$p_{11}$	$p_{11}=\left(cas9,gRNA_{g_{5}}^{delete}\right)$	$p_{s_{9}}$	$p_{s_{9}}=\left(gRNA_{g_{6}}^{template}\right)$
$p_{12}$	$p_{12}=\left(cas9,gRNA_{g_{5}}^{delete}\right)$	$p_{s_{16}}$	$p_{s_{16}}=\left(gRNA_{g_{4}}^{template}\right)$
$p_{13}$	$p_{13}=\left(cas9,gRNA_{g_{2}}^{delete}\right)$	$p_{s_{17}}$	$p_{s_{17}}=\left(gRNA_{g_{2}}^{template}\right)$
$p_{14}$	$p_{14}=\left(cas9,gRNA_{g_{5}}^{delete}\right)$	$p_{s_{20}}$	$p_{s_{20}}=\left(gRNA_{g_{0}}^{template}\right)$
$p_{15}$	$p_{15}=\left(cas9,gRNA_{g_{3}}^{delete}\right)$	$p_{s_{21}}$	$p_{s_{21}}=\left(gRNA_{g_{3}}^{template}\right)$
$p_{null}$	$\left(Pro_{Rap}^{Rel}\right)$	$p_{s_{h}}$	$p_{s_{h}}=\left(Pro_{Rap}^{Rel}\right)$

**Table 2:** Rules in each bacterium of system Π.

Sim.	Rules	Bac.
$l_{0}$	$\left(\mathrm{ATP}-p_{0}p_{null},b_{1}/b_{2},\mathrm{ATP}-p_{0}p_{null}\right)$	$b_{1}$
	$p_{0}\times \left(g_{1},g_{m}\right)$, $\left(\mathrm{ATP}-p_{null},b_{2}/b_{1},\mathrm{ATP}-p_{1}p_{s_{1}}\right)$, $\left(\mathrm{ATP}-p_{0}p_{null},b_{2}/b_{1},\mathrm{ATP}-p_{2}p_{s_{2}}\right)$	$b_{2}$
$l_{1}$	$\left(\mathrm{ATP}-p_{1}p_{s_{1}},b_{1}/b_{2},\mathrm{ATP}-p_{1}p_{s_{1}}\right)$	$b_{1}$
	$p_{1}\times \left(g_{7},g_{m}\right)$, $\left(\mathrm{ATP}-p_{s_{1}},b_{2}/b_{1},\mathrm{ATP}-p_{0}p_{null}\right)$	$b_{2}$
$l_{2}$	$\left(\mathrm{ATP}-p_{2}p_{s_{2}},b_{1}/b_{2},\mathrm{ATP}-p_{2}p_{s_{2}}\right)$	$b_{1}$
	$p_{2}\times \left(g_{6},g_{m}\right)$, $\left(\mathrm{ATP}-p_{s_{2}},b_{2}/b_{1},\mathrm{ATP}-p_{3}p_{null}\right)$	$b_{2}$
$l_{3}$	$\left(\mathrm{ATP}-p_{3}p_{null},b_{1}/b_{2},\mathrm{ATP}-p_{3}p_{null}\right)$	$b_{1}$
	$p_{3}\times \left(g_{5},g_{m}\right)$, $\left(\mathrm{ATP}-p_{null},b_{2}/b_{1},\mathrm{ATP}-p_{2}p_{s_{2}}\right)$, $\left(\mathrm{ATP}-p_{3}p_{null},b_{2}/b_{1},\mathrm{ATP}-p_{4}p_{null}\right)$	$b_{2}$
$l_{4}$	$\left(\mathrm{ATP}-p_{4}p_{null},b_{1}/b_{2},\mathrm{ATP}-p_{4}p_{null}\right)$	$b_{1}$
	$p_{4}\times \left(g_{6},g_{m}\right)$, $\left(\mathrm{ATP}-p_{null},b_{2}/b_{1},\mathrm{ATP}-p_{5}p_{s_{5}}\right)$, $\left(\mathrm{ATP}-p_{4}p_{null},b_{2}/b_{1},\mathrm{ATP}-p_{3}p_{null}\right)$	$b_{2}$
$l_{5}$	$\left(\mathrm{ATP}-p_{5}p_{s_{5}},b_{1}/b_{2},\mathrm{ATP}-p_{5}p_{s_{5}}\right)$	$b_{1}$
	$p_{1}\times \left(g_{5},g_{m}\right)$, $\left(\mathrm{ATP}-p_{s_{5}},b_{2}/b_{1},\mathrm{ATP}-p_{6}p_{null}\right)$	$b_{2}$
$l_{6}$	$\left(\mathrm{ATP}-p_{6}p_{null},b_{1}/b_{2},\mathrm{ATP}-p_{6}p_{null}\right)$	$b_{1}$
	$p_{6}\times \left(g_{7},g_{m}\right)$, $\left(\mathrm{ATP}-p_{null},b_{2}/b_{1},\mathrm{ATP}-p_{7}p_{s_{7}}\right)$, $\left(\mathrm{ATP}-p_{6}p_{null},b_{2}/b_{1},\mathrm{ATP}-p_{8}p_{null}\right)$	$b_{2}$
$l_{7}$	$\left(\mathrm{ATP}-p_{7}p_{s_{7}},b_{1}/b_{2},\mathrm{ATP}-p_{7}p_{s_{7}}\right)$	$b_{1}$
	$p_{7}\times \left(g_{1},g_{m}\right)$, $\left(\mathrm{ATP}-p_{4}p_{null},b_{2}/b_{1},\mathrm{ATP}-p_{4}p_{null}\right)$	$b_{2}$
$l_{8}$	$\left(\mathrm{ATP}-p_{8}p_{null},b_{1}/b_{2},\mathrm{ATP}-p_{8}p_{null}\right)$	$b_{1}$
	$p_{8}\times \left(g_{6},g_{m}\right)$, $\left(\mathrm{ATP}-p_{null},b_{2}/b_{1},\mathrm{ATP}-p_{9}p_{s_{9}}\right)$, $\left(\mathrm{ATP}-p_{0}p_{null},b_{2}/b_{1},\mathrm{ATP}-p_{0}p_{null}\right)$	$b_{2}$
$l_{9}$	$\left(\mathrm{ATP}-p_{9}p_{s_{9}},b_{1}/b_{2},\mathrm{ATP}-p_{9}p_{s_{9}}\right)$	$b_{1}$
	$p_{9}\times \left(g_{6},g_{m}\right)$, $\left(\mathrm{ATP}-p_{10}p_{null},b_{2}/b_{1},\mathrm{ATP}-p_{10}p_{null}\right)$	$b_{2}$
$l_{10}$	$\left(\mathrm{ATP}-p_{10}p_{null},b_{1}/b_{2},\mathrm{ATP}-p_{10}p_{null}\right)$	$b_{1}$
	$p_{10}\times \left(g_{4},g_{m}\right)$, $\left(\mathrm{ATP}-p_{0}p_{null},b_{2}/b_{1},\mathrm{ATP}-p_{0}p_{null}\right)$, $\left(\mathrm{ATP}-p_{10}p_{null},b_{2}/b_{1},\mathrm{ATP}-p_{11}p_{null}\right)$	$b_{2}$
$l_{11}$	$\left(\mathrm{ATP}-p_{10}p_{null},b_{1}/b_{2},\mathrm{ATP}-p_{11}p_{null}\right)$	$b_{1}$
	$p_{11}\times \left(g_{5},g_{m}\right)$, $\left(\mathrm{ATP}-p_{null},b_{2}/b_{1},\mathrm{ATP}-p_{12}p_{null}\right)$, $\left(\mathrm{ATP}-p_{11}p_{null},b_{2}/b_{1},\mathrm{ATP}-p_{13}p_{null}\right)$	$b_{2}$
$l_{12}$	$\left(\mathrm{ATP}-p_{12}p_{null},b_{1}/b_{2},\mathrm{ATP}-p_{12}p_{null}\right)$	$b_{1}$
	$p_{12}\times \left(g_{5},g_{m}\right)$, $\left(\mathrm{ATP}-p_{null},b_{2}/b_{1},\mathrm{ATP}-p_{14}p_{null}\right)$, $\left(\mathrm{ATP}-p_{12}p_{null},b_{2}/b_{1},\mathrm{ATP}-p_{15}p_{null}\right)$	$b_{2}$
$l_{13}$	$\left(\mathrm{ATP}-p_{13}p_{null},b_{1}/b_{2},\mathrm{ATP}-p_{13}p_{null}\right)$	$b_{1}$
	$p_{13}\times \left(g_{2},g_{m}\right)$, $\left(\mathrm{ATP}-p_{null},b_{2}/b_{1},\mathrm{ATP}-p_{18}p_{null}\right)$, $\left(\mathrm{ATP}-p_{13}p_{null},b_{2}/b_{1},\mathrm{ATP}-p_{19}p_{null}\right)$	$b_{2}$
$l_{14}$	$\left(\mathrm{ATP}-p_{14}p_{null},b_{1}/b_{2},\mathrm{ATP}-p_{14}p_{null}\right)$	$b_{1}$
	$p_{14}\times \left(g_{5},g_{m}\right)$, $\left(\mathrm{ATP}-p_{null},b_{2}/b_{1},\mathrm{ATP}-p_{16}p_{s_{16}}\right)$, $\left(\mathrm{ATP}-p_{14}p_{null},b_{2}/b_{1},\mathrm{ATP}-p_{17}p_{s_{17}}\right)$	$b_{2}$
$l_{15}$	$\left(\mathrm{ATP}-p_{15}p_{null},b_{1}/b_{2},\mathrm{ATP}-p_{15}p_{null}\right)$	$b_{1}$
	$p_{15}\times \left(g_{3},g_{m}\right)$, $\left(\mathrm{ATP}-p_{null},b_{2}/b_{1},\mathrm{ATP}-p_{18}p_{null}\right)$, $\left(\mathrm{ATP}-p_{15},b_{2}/b_{1},\mathrm{ATP}-p_{20}p_{s_{20}}\right)$	$b_{2}$
$l_{16}$	$\left(\mathrm{ATP}-p_{16}p_{s_{16}},b_{1}/b_{2},\mathrm{ATP}-p_{16}p_{s_{16}}\right)$	$b_{1}$
	$p_{16}\times \left(g_{4},g_{m}\right)$, $\left(\mathrm{ATP}-p_{s_{16}},b_{2}/b_{1},\mathrm{ATP}-p_{11}p_{null}\right)$	$b_{2}$
$l_{17}$	$\left(\mathrm{ATP}-p_{17}p_{s_{17}},b_{1}/b_{2},\mathrm{ATP}-p_{17}p_{s_{17}}\right)$	$b_{1}$
	$p_{17}\times \left(g_{2},g_{m}\right)$, $\left(\mathrm{ATP}-p_{s_{17}},b_{2}/b_{1},\mathrm{ATP}-p_{21}p_{s_{21}}\right)$	$b_{2}$
$l_{18}$	$\left(\mathrm{ATP}-p_{18}p_{null},b_{1}/b_{2},\mathrm{ATP}-p_{18}p_{null}\right)$	$b_{1}$
	$p_{18}\times \left(g_{4},g_{m}\right)$, $\left(\mathrm{ATP}-p_{null},b_{2}/b_{1},\mathrm{ATP}-p_{0}p_{null}\right)$, $\left(\mathrm{ATP}-p_{18}p_{null},b_{2}/b_{1},\mathrm{ATP}-p_{h}p_{s_{h}}\right)$	$b_{2}$
$l_{19}$	$\left(\mathrm{ATP}-p_{19}p_{null},b_{1}/b_{2},\mathrm{ATP}-p_{19}p_{null}\right)$	$b_{1}$
	$p_{19}\times \left(g_{3},g_{m}\right)$, $\left(\mathrm{ATP}-p_{null},b_{2}/b_{1},\mathrm{ATP}-p_{0}p_{null}\right)$, $\left(\mathrm{ATP}-p_{15},b_{2}/b_{1},\mathrm{ATP}-p_{18}p_{null}\right)$	$b_{2}$
$l_{20}$	$\left(\mathrm{ATP}-p_{20}p_{s_{20}},b_{1}/b_{2},\mathrm{ATP}-p_{20}p_{s_{20}}\right)$	$b_{1}$
	$p_{20}\times \left(g_{0},g_{m}\right)$, $\left(\mathrm{ATP}-p_{s_{20}},b_{2}/b_{1},\mathrm{ATP}-p_{0}p_{null}\right)$	$b_{2}$
$l_{21}$	$\left(\mathrm{ATP}-p_{21}p_{s_{21}},b_{1}/b_{2},\mathrm{ATP}-p_{21}p_{s_{21}}\right)$	$b_{1}$
	$p_{0}\times \left(g_{3},g_{m}\right)$, $\left(\mathrm{ATP}-p_{s_{21}},b_{2}/b_{1},\mathrm{ATP}-p_{18}p_{null}\right)$	$b_{2}$
$l_{h}$	$\left(\mathrm{ATP}-p_{h}p_{s_{h}},b_{1}/b_{2},\mathrm{ATP}-p_{h}p_{s_{h}}\right)$	
